# Data on the main working conditions with influence on the development of hearing loss amongst the occupational population in Spain

**DOI:** 10.1016/j.dib.2018.08.054

**Published:** 2018-09-05

**Authors:** Jesús P. Barrero, S. García-Herrero, M.A. Mariscal, J.M. Gutiérrez

**Affiliations:** a University of Burgos, Spain; b University of Cantabria, Spain

## Abstract

Obtaining reliable and objective data on certain working conditions is necessary to analyse the causes and variables that can influence the development of hearing loss amongst the working population. Objective occupational data have been collected from a heterogeneous sample of 1418 workers in Spain, see “How activity type, time on the job and noise level on the job affect the hearing of the working population. Using Bayesian networks to predict the development of hipoacusia” (Barrero et al., 2018) [1]. Among the main factors analysed are the noise levels to which these workers are exposed, measured at their respective workstations, and the assessment of their hearing status, evaluated by audiometric medical tests. These factors provide information to predict the development of hypoacusia.

**Specifications Table**

**Table:** 

Subject area	Population health
Specific subject area	Occupational health and safety
Type of data	Tables, figures
How data were acquired	Sound level meters, noise dosimeters and audiometric medical testing
Data format	Filtered, processed
Experimental factors	Occupational conditions from 1418 workers.
Experimental features	Noise levels exposure and audiometric medical tests
Data location	Spain
Data accessibility	Data is with this article.
Related research article	Jesús P.Barrero, Susana García-Herrero, Miguel A. Mariscal and J.M.Gutierrez, How activity type, time on the job and noise level on the job affect the hearing of the working population. Using Bayesian networks to predict the development of hypoacusia. Safety Science, Volume 110, Part A, December 2018, pp. 1–12. https://doi.org/10.1016/j.ssci.2018.07.011.

**Value of data**

• The dataset shows the average noise levels to which workers from different sectors of activity are exposed and can be used to match data from other countries or sectors of activity.
• The dataset can be used to show different aspects of occupational exposure to noise, such as daily noise exposure (hours), number of years in the workplace, noise exposure in previous employment, noise protection system based on hearing protection or time limitation.
• The dataset can be useful for researchers to see the results of the audiometric studies carried out on workers. The workers hearing health has been compared to international indices of auditory assessment.
• The dataset provides information for future health and safety at work studies, with special interest for Health and Safety Technical Experts and medical professionals.

## 1 Data

Medical and occupational environment data were collected over a period of approximately two years from a sample of 1418 workers from different activity sectors, ages and nationalities, who were working in the provinces of Burgos and Valladolid, Spain. Ingemédica S.L., an occupational health and safety consultancy, has collaborated with the University of Burgos to collect the data.

The dataset was designed to answer the basic questions of how and why some workers develop hypoacusia. The variables are classified as demographic and personal factors (meaning those that characterise a specific population) occupational factors (those related to the working conditions in different companies) and non-occupational factors (those that are manifested outside the work environment) [1].

Data from 1418 workers have been obtained including demographic/personal data (age, gender, height, weight, nationality, blood pressure, etc), data on occupational factors (the type of sector or activity of the company where these people work, job title, noise levels, daily exposure, number of years at work, the use or not of hearing protection, whether or not there is any limitation of temporary exposure to noise, occupational exposure to noise in previous employment, exposure to ototoxic agents) and data on non occupational factors (pre-existing auditive diseases, and the use of medicines that may have otic side effects).

All the data were anonymised and collected with the consent of the companies and individuals involved.

**Fig. 1:**
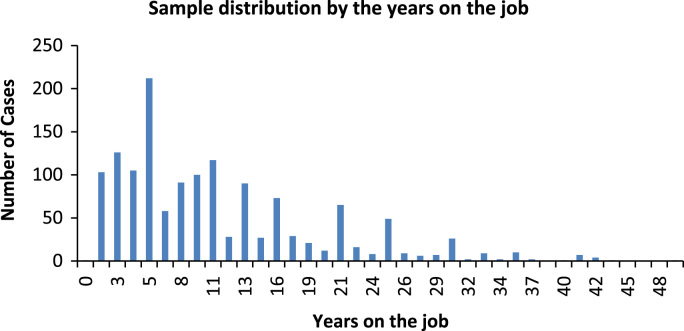
Sample distribution by the years on the job. *Source*: Compiled by authors.

**Fig. 2:**
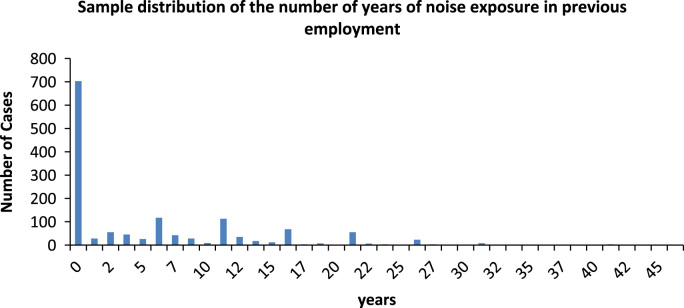
Sample distribution of the number of years of noise exposure in previous employment. *Source*: Compiled by authors.

See Table 1, Table 2, Table 3, Table 4, Table 5, Table 6, Table 7, Table 8, Table 9, Table 10, Table 11, Table 12, Table 13, Table 14, Table 15, Table 16, Table 17, Table 18, Table 19, Table 20, Table 21, Table 22, Table 23, Table 24, Table 25, Table 26, Table 27, Table 28, Table 29, Table 30, Table 31, Table 32 and Fig. 1, Fig. 2.

## 2 Experimental design, materials and methods

The necessary data has been achieved through two main lines of work. The first, focused on obtaining the data referring to the noise levels at the workstations, has been carried out using sound level meters and noise dosimeters. These measurements have been made by qualified occupational hygienists. The second line of work was consisted of carrying out medical tests which included audiometries and questionnaires. The questionnaire, based on Occupational Health Surveillance Protocols, was developed by the Department of Health and Welfare of the Junta de Castilla y León and authorised by the Ministry of Health and Consumer Affairs of Spain [2]. In compliance with the Health Surveillance Protocols and the current Spanish regulations on Health and Safety at work [3], [4] the audiometric tests were carried out by specialised personnel (occupational physicians and nurses) using audiometers and soundproofed cabins.

This section considers the frequencies and categories associated with the main occupational factors selected as influential in the development of hearing loss:

### 2.1 Noise level

#### 2.1.1 Noise level of the sample

The results of the noise levels were divided into four groups, in keeping with Spanish Royal Decree 286/2006. The groups are: Low (LAeq.d < 80 dB and LPeak < 135 dB), Moderate (LAeq.d ≥ 80 dB <85 dB and LPeak ≥ 135 dB < 137 dB), High (LAeq.d ≥ 85 dB < 87 dB and LPeak ≥ 137 dB < 140 dB) and Very High (LAeq.d ≥ 87 dB and LPeak ≥ 140 dB). The percentage frequency of each would be 30.68%, 46.54%, 7,69% and 15.09%, respectively.

#### 2.1.2 Noise level by activity sector

The sample has been divided into the traditional economic sectors which are: Construction, Agriculture/Livestock, Industry and Services. The percentage frequency of each would be 54.16%, 0.35%, 22.85% and 22.64%, respectively.

• **Sector: Construction.**

The noise level distribution for the sample related to the construction sector can be seen in Table 1.

• **Sector: Agriculture/Livestock**

**Table 1:** Noise level distribution in the construction sector.

Group	Noise level sector: construction (dB)	# of cases	Frequency %
1. Low	L_Aeq.d_ < 80 and L_Peak_ < 135	99	12.89
2. Moderate	L_Aeq.d_ ≥ 80 < 85 and L_Peak_ ≥ 135 < 137	484	63.02
3. High	L_Aeq.d_ ≥85<87 and L_Peak_ ≥ 137 < 140	51	17.45
4. Very high	L_Aeq.d_ ≥87 and L_Peak_ ≥ 140	134	6.64
	Total	768	100

Table 2 shows the noise level distribution related to the sector of Agriculture/Livestock.

• **Sector: Industry**

**Table 2:** Noise level distribution in Agriculture/Livestock sector.

Group	Noise level sector: agriculture/livestock (dB)	# of cases	Frequency %
1. Low	L_Aeq.d_ < 80 and L_Peak_ < 135	5	100
2. Moderate	L_Aeq.d_ ≥ 80< 85 and L_Peak_ ≥ 135 < 137	0	0.00
3. High	L_Aeq.d_ ≥ 85 < 87 and L_Peak_ ≥ 137 < 140	0	0.00
4. Very high	L_Aeq.d_ ≥ 87 and L_Peak_ ≥ 140	0	0.00
	Total	5	100

Noise level distribution for the industry related sample can be seen in Table 3.

• **Sector: Services**

**Table 3:** Noise level distribution in the industry sector.

Group	Noise level sector: industry (dB)	# of cases	Frequency %
1. Low	L_Aeq.d_ < 80 and L_Peak_ < 135	119	36.76
2. Moderate	L_Aeq.d_ ≥ 80 < 85 and L_Peak_ ≥ 135 < 137	98	30.25
3. High	L_Aeq.d_ ≥ 85 < 87 and L_Peak_ ≥ 137 < 140	55	16.98
4. Very high	L_Aeq.d_ ≥ 87 and L_Peak_ ≥ 140	52	16.05
	Total	324	100

Noise level distribution for the sample related to the Services sector can be seen in Table 4.

**Table 4:** Noise level distribution in the services sector.

Group	Noise level sector: services (dB)	# of cases	Frequency %
1. Low	L_Aeq.d_ < 80 and L_Peak_ < 135	212	66.04
2. Moderate	L_Aeq.d_ ≥80 < 85 and L_Peak_ ≥ 135 < 137	78	24.30
3. High	L_Aeq.d_ ≥ 85 < 87 and L_Peak_ ≥ 137 < 140	3	0.93
4. Very high	L_Aeq.d_ ≥ 87 and L_Peak_ ≥ 140	28	8.72
	Total	321	100

#### 2.1.3 Noise level by job title

Table 5 shows how the sample is distributed according to the different jobs analysed.

• **Job title: Administration**

**Table 5:** Distribution by job title.

Group	Job title	# of cases	Frequency %
1	Administration	106	7.48
2	Warehouse operative	18	1.27
3	Carpenter	18	1.27
4	Sales representative	27	1.90
5	Driver (vehicles, construction machinery, forklift)	209	14.74
6	Shop assistant/Recepcionist	29	2.05
7	Nurse/Assistant nurse	5	0.35
8	Electrician/Technician/Installer	46	3.24
9	Section Manager/Site manager	76	5.36
10	Plumber	33	2.33
11	Livestock farmer	3	0.21
12	General Manager/Director	14	0.99
13	Engineer/Architect	72	5.08
14	Gardener	42	2.96
15	Cleaner	7	0.49
16	Maintenance worker	14	0.99
17	Machine operator (lathe, milling machine)	23	1.62
18	Mechanic	60	4.23
19	Assembler	11	0.78
20	Services (Waiter, hairdresser…)	30	2.12
21	Construction worker	304	21.44
22	Industry worker	2	0.14
23	Food production worker	23	1.62
24	Concrete Production worker	23	1.62
25	Manufacturing industry worker	20	1.41
26	Paper production worker	23	1.62
27	Chemical production worker	92	6.49
28	Delivery driver	10	0.71
29	Welder	75	5.29
30	Security guard	3	0.21
	Total	1418	100

Table 6 shows the noise level distribution for the administration personnel.

• **Job title: Warehouse operative**

**Table 6:** Noise level distribution for the administration personnel.

Group	Noise level workstation administration (dB)	# of cases	Frequency %
1. Low	L_Aeq.d_ < 80 and L_Peak_ < 135	106	100
2. Moderate	L_Aeq.d_ ≥ 80 < 85 and L_Peak_ ≥ 135 < 137	0	0.00
3. High	L_Aeq.d_ ≥ 85 < 87 and L_Peak_ ≥ 137 < 140	0	0.00
4. Very high	L_Aeq.d_ ≥ 87 and L_Peak_ ≥ 140	0	0.00
	Total	106	100

The noise level distribution for warehouse operatives is presented in Table 7.

• **Job title: Carpenter**

**Table 7:** Noise level distribution for the warehouse operatives.

Group	Noise level workstation warehouse in dB	# of cases	Frequency %
1. Low	L_Aeq.d_ < 80 and L_Peak_ < 135	18	100
2. Moderate	L_Aeq.d_ ≥ 80 < 85 and L_Peak_ ≥ 135 < 137	0	0.00
3. High	L_Aeq.d_ ≥ 85 < 87 and L_Peak_ ≥ 137 < 140	0	0,00
4. Very high	L_Aeq.d_ ≥ 87 and L_Peak_ ≥ 140	0	0,00
	Total	18	100

Table 8 shows the noise level distribution for the carpenter׳s workstation.

• **Job title: Sales representative**

**Table 8:** Noise level distribution for the carpenter׳s workstation.

Group	Noise level carpenter׳s workstation (dB)	# of cases	Frequency %
1. Low	L_Aeq.d_ < 80 and L_Peak_ < 135	0	0.00
2. Moderate	L_Aeq.d_ ≥ 80 < 85 and L_Peak_ ≥ 135 < 137	4	22.22
3. High	L_Aeq.d_ ≥ 85 < 87 and L_Peak_ ≥ 137 < 140	0	0,00
4. Very high	L_Aeq.d_ ≥ 87 and L_Peak_ ≥ 140	14	72.78
	Total	18	100

The noise level distribution for the sales representative position can be seen in Table 9.

• **Job title: Driver**

**Table 9:** Noise level distribution for the sales manager position.

Group	Noise level workstation sales representative in dB	# of cases	Frequency %
1. Low	L_Aeq.d_ < 80 and L_Peak_ < 135	25	92,59
2. Moderate	L_Aeq.d_ ≥ 80 < 85 and L_Peak_ ≥ 135 < 137	1	3,70
3. High	L_Aeq.d_ ≥ 85 < 87 and L_Peak_ ≥ 137 < 140	0	0,00
4. Very high	L_Aeq.d_ ≥ 87 and L_Peak_ ≥ 140	1	3,70
	Total	27	100

Table 10 shows the noise levels for the driver position.

• **Job title: Shop assistant/receptionist**

**Table 10:** Noise level distribution for the driver position.

Group	Noise level driver (vehicles, construction machinery, etc) in dB	# of cases	Frequency %
1. Low	L_Aeq.d_ < 80 and L_Peak_ < 135	10	4.78
2. Moderate	L_Aeq.d_ ≥ 80 < 85 and L_Peak_ ≥ 135 < 137	52	24.88
3. High	L_Aeq.d_ ≥ 85 < 87 and L_Peak_ ≥ 137 < 140	19	9.09
4. Very high	L_Aeq.d_ ≥ 87 and L_Peak_ ≥140	128	61.24
	Total	209	100

The noise level distribution for the shop assistant/receptionist is shown in Table 11.

• **Job title: Nurse/Assistant nurse**

**Table 11:** Noise level distribution for the shop assistant/receptionist.

Group	Noise level shop assistant/recepcionist (dB)	# of cases	Frequency %
1. Low	L_Aeq.d_ < 80 and L_Peak_ < 135	25	86.21
2. Moderate	L_Aeq.d_ ≥ 80 < 85 and L_Peak_ ≥ 135 < 137	4	13.79
3. High	L_Aeq.d_ ≥ 85 < 87 and L_Peak_ ≥ 137 < 140	0	0.00
4. Very high	L_Aeq.d_ ≥ 87 and L_Peak_ ≥ 140	0	0.00
	Total	29	100

Table 12 shows the noise level distribution for nurse/assistant nurse position.

• **Job title: Electrician/Technician/Installer**

**Table 12:** Noise level distribution for nurse/assistant nurse position.

Group	Noise level workstation nurse/assistant nurse (dB)	# of cases	Frequency %
1. Low	L_Aeq.d_ < 80 and L_Peak_ < 135	5	100
2. Moderate	L_Aeq.d_ ≥ 80 < 85 and L_Peak_ ≥ 135 < 137	0	0.00
3. High	L_Aeq.d_ ≥ 85 < 87 and L_Peak_ ≥ 137 < 140	0	0.00
4. Very high	L_Aeq.d_ ≥ 87 and L_Peak_ ≥ 140	0	0.00
	Total	5	100

The noise level distribution for the electrician, technician and installer workstations is shown in Table 13.

• **Job title: Section Manager/Site manager**

**Table 13:** Noise level distribution for the Electrician/Technician/Installer workstations.

Group	Noise level workstation Electrician/Technician/Iinstaller in dB	# of cases	Frequency %
1. Low	L_Aeq.d_ < 80 and L_Peak_ < 135	29	63.04
2. Moderate	L_Aeq.d_ ≥ 80 < 85 and L_Peak_ ≥ 135 < 137	17	36.96
3. High	L_Aeq.d_ ≥ 85 < 87 and L_Peak_ ≥ 137 < 140	0	0.00
4. Very high	L_Aeq.d_ ≥ 87 and L_Peak_ ≥ 140	0	0.00
	Total	46	100

Table 14 shows the noise level distribution for the workstation: Manager/Site manager.

• **Job title: Plumber**

**Table 14:** Noise level distribution for the Manager/Site manager workstation.

Group	Noise level workstation Manager/Site manager (dB)	# of cases	Frequency %
1. Low	L_Aeq.d_ < 80 and L_Peak_ < 135	5	6.58
2. Moderate	L_Aeq.d_ ≥ 80 < 85 and L_Peak_ ≥ 135 < 137	50	65.79
3. High	L_Aeq.d_ ≥ 85 < 87 and L_Peak_ ≥ 137 < 140	6	7.89
4. Very high	L_Aeq.d_ ≥ 87 and L_Peak_ ≥ 140	15	19.74
	Total	76	100

The noise level distribution for the plumber׳s workstation can be seen in Table 15.

• **Job title: General Manager/Director**

**Table 15:** Noise level distribution for plumber workstation.

Group	Noise level workstation plumber in dB	# of cases	Frequency %
1. Low	L_Aeq.d_ < 80 and L_Peak_ < 135	19	57.58
2. Moderate	L_Aeq.d_ ≥ 80 < 85 and L_Peak_ ≥ 135 < 137	14	42.42
3. High	L_Aeq.d_ ≥ 85 < 87 and L_Peak_ ≥ 137 < 140	0	0.00
4. Very high	L_Aeq.d_ ≥ 87 and L_Peak_ ≥ 140	0	0.00
	Total	33	100

The noise level distribution for the General Manager/Director is shown in Table 16.

• **Job title: Engineer/Architect**

**Table 16:** Noise level distribution for General Manager/Director.

Group	Noise level workstation General Manager/Director (dB)	# of cases	Frequency %
1. Low	L_Aeq.d_ < 80 and L_Peak_ < 135	11	78.57
2. Moderate	L_Aeq.d_ ≥ 80 < 85 and L_Peak_ ≥ 135 < 137	2	14.29
3. High	L_Aeq.d_ ≥ 85 < 87 and L_Peak_ ≥ 137 < 140	1	7.14
4. Very high	L_Aeq.d_ ≥ 87 and L_Peak_ ≥ 140	0	0.00
	Total	14	100

Table 17 shows the noise level distribution for the Engineer/Architect position.

• **Job title: Gardener**

**Table 17:** Noise level distribution for Engineer/Architect position.

Group	Noise level workstation Engineer/Architect (dB)	# of cases	Frequency %
1. Low	L_Aeq.d_ < 80 and L_Peak_ < 135	66	91.67
2. Moderate	L_Aeq.d_ ≥ 80 < 85 and L_Peak_ ≥ 135 < 137	6	8.33
3. High	L_Aeq.d_ ≥ 85 < 87 and L_Peak_ ≥ 137 < 140	0	0.00
4. Very high	L_Aeq.d_ ≥ 87 and L_Peak_ ≥ 140	0	0.00
	Total	72	100

The noise level distribution for the gardener position is shown in Table 18.

• **Job title: Cleaner**

**Table 18:** Noise level distribution for gardener position.

Group	Noise level gardener (dB)	# of cases	Frequency %
1. Low	L_Aeq.d_ < 80 and L_Peak_ < 135	0	0.00
2. Moderate	L_Aeq.d_ ≥ 80 < 85 and L_Peak_ ≥ 135 < 137	42	100
3. High	L_Aeq.d_ ≥ 85 < 87 and L_Peak_ ≥ 137 < 140	0	0.00
4. Very high	L_Aeq.d_ ≥ 87 and L_Peak_ ≥ 140	0	0.00
	Total	42	100

Table 19 shows the noise level distribution for the cleaners.

• **Job title: Production operator**

**Table 19:** Noise level distribution for the cleaners position.

Group	Noise level cleaner (dB)	# of cases	Frequency %
1. Low	L_Aeq.d_ < 80 and L_Peak_ < 135	4	57.14
2. Moderate	L_Aeq.d_ ≥ 80 < 85 and L_Peak_ ≥ 135 < 137	3	42.86
3. High	L_Aeq.d_ ≥ 85 < 87 and L_Peak_ ≥ 137 < 140	0	0.00
4. Very high	L_Aeq.d_ ≥ 87 and L_Peak_ ≥ 140	0	0.00
	Total	7	100

The job title “production operator” includes several job titles, e.g. construction worker, industry worker, food production worker, concrete production worker, manufacturing industry worker, paper production worker and chemical production worker. Table 20 shows the noise level distribution for the production operator.

• **Job title: Delivery driver**

**Table 20:** Noise level distribution for production operator.

Group	Noise level production operator (dB)	# of cases	Frequency %
1	L_Aeq.d_ < 80 and L_Peak_ < 135	94	15.04
2	L_Aeq.d_ ≥ 80 < 85 and L_Peak_ ≥ 135 < 137	394	63.04
3	L_Aeq.d_ ≥ 85 < 87 and L_Peak_ ≥ 137 < 140	83	13.28
4	L_Aeq.d_ ≥ 87 and L_Peak_ ≥ 140	54	8.64
	Total	625	100

Table 21 shows the noise level distribution for the delivery driver position.

• **Job title: Welder**

**Table 21:** Noise level distribution for the delivery driver position.

Group	Noise level workstation delivery driver in dB	# of cases	Frequency %
1	L_Aeq.d_ < 80 and L_Peak_ < 135	10	100
2	L_Aeq.d_ ≥ 80 < 85 and L_Peak_ ≥ 135 < 137	0	0.00
3	L_Aeq.d_ ≥ 85 < 87 and L_Peak_ ≥ 137 < 140	0	0.00
4	L_Aeq.d_ ≥ 87 and L_Peak_ ≥ 140	0	0.00
	Total	10	100

Table 22 shows the noise level distribution for the welder workstation.

● **Job title: Security guard**

**Table 22:** Noise level distribution for welder operator position.

Group	Noise level welder (dB)	# of cases	Frequency %
1. Low	L_Aeq.d_ < 80 and L_Peak_ < 135	0	0.00
2. Moderate	L_Aeq.d_ ≥ 80 < 85 and L_Peak_ ≥ 135 < 137	73	97.33
3. High	L_Aeq.d_ ≥ 85 < 87 and L_Peak_ ≥ 137 < 140	0	0.00
4. Very high	L_Aeq.d_ ≥ 87 and L_Peak_ ≥ 140	2	2.67
	Total	75	100

Table 23 shows the noise level distribution for the security guard position.

**Table 23:** Noise level distribution for the security guard position.

Group	Noise level security guard (dB)	# of cases	Frequency %
1. Low	L_Aeq.d_ < 80 and L_Peak_ < 135	3	100
2. Moderate	L_Aeq.d_ ≥ 80 < 85 and L_Peak_ ≥ 135 < 137	0	0.00
3. High	L_Aeq.d_ ≥ 85 < 87 and L_Peak_ ≥ 137 < 140	0	0.00
4. Very high	L_Aeq.d_ ≥ 87 and L_Peak_ ≥ 140	0	0.00
	Total	3	100

### 2.2 Exposure

#### 2.2.1 Daily noise exposure (hours)

Table 24 shows the daily noise exposure in hours.

**Table 24:** Daily noise exposure (hours).

Group	Daily noise exposure (h)	# of cases	Frequency %
1	< 8	52	3.67
2	8	1129	79.62
3	> 8	237	16.71
	Total	1418	100

#### 2.2.2 Years on the job

Fig. 1 shows the distribution of the sample according to the number of years the employees have been at their work locations. The average is 10.2 years, with a minimum value of 0 years and a maximum of 49 years.

This variable has been discretized as shown in Table 25.

**Table 25:** Sample distribution by years on the job.

Group	Years on the job	# of cases	Frequency %
1	< 3	230	16.22
2	≥ 3 <6	317	22.36
3	≥ 6 <10	249	17.56
4	≥ 10 <16	335	23.62
5	≥ 16	287	20.00
	Total	1418	100

#### 2.2.3 Number of years of noise exposure in previous jobs

Fig. 2 shows the sample distribution according to the number of years of noise exposure in previous employment. The average is in 5.2 years, with a minimum value of 0 years and a maximum of 46 years.

Table 26 shows the sample distribution according to the number of years in previous employment.

**Table 26:** Sample distribution of the number of years of noise exposure in previous employment.

Group	Number of years of noise exposure in previous employment	# of cases	Frequency %
1	0	703	49.58
2	≥ 0 <3	83	5.85
3	≥ 3 <6	159	11.21
4	≥ 6 <10	108	7.62
5	≥ 10	365	26.00
	Total	1418	100

#### 2.2.4 Noise protection system based on hearing protection

The sample is divided into two unique groups, depending on whether or not the worker uses hearing protection as a noise protection system. The results of the distribution can be seen in Table 27.

**Table 27:** Distribution of the sample by the use of hearing protection.

Group	Use of hearing protection	# of cases	Frequency %
1	No	986	69.53
2	Yes	432	30.47
	Total	1418	100

#### 2.2.5 Noise protection system based on time limits

Table 28 shows the sample distribution by noise protection based on time limits.

**Table 28:** Noise protection system based on time limits.

Group	Noise protection system based on time limits	# of cases	Frequency %
1	No	1261	88.93
2	Yes	157	11.07
	Total	1418	100

#### 2.2.6 Occupational exposure to ototoxic agents

The sample has been divided into two groups, depending on whether the worker has been exposed to ototoxic agents or not, such as: carbon monoxide, lead, benzene and mercury. The results of their distribution in the two groups can be seen in Table 29.

**Table 29:** Sample distribution by exposure to ototoxic agents.

Group	Occupational exposure to ototoxic agents	# of cases	Frequency %
1	No	1338	94.36
2	Yes	80	5.64
	Total	1418	100

### 2.3 The sample׳s auditory health. Results of the audiometric study

Below are the overall results of the hearing tests performed. Sal, ELI and Global Hearing Loss Percentages have been used to analyse these results.

#### 2.3.1 SAL index

The SAL index (Speech Average Loss), evaluates the conversation frequencies in 500 Hz, 1000 Hz and 2000 Hz to then perform the arithmetic mean of the hearing loss in decibels of those frequencies. The SAL index classifies the results from A to G depending on the worsening of hearing; SAL-A meaning both ears are within normal limits and SAL-G total deafness [2]. Table 30 shows the distribution of the sample in accordance with the SAL index.

**Table 30:** Sample distribution according to SAL index.

Group	SAL index	# of cases	Frequency %
1	A. Normal hearing	972	68.55
2	B. Nearly normal hearing	419	29.55
3	C. Slight deterioration	24	1.69
4	D. Serious deterioration	1	0.04
5	E. Severe deterioration	2	0.07
6	F. Heavy deterioration	0	0
7	G. Total deafness in both ears	0	0
	Total	1418	100

#### 2.3.2 ELI index

The ELI index (Early Loss Index) is calculated by subtracting a correction value for presbycusis from the loss in the frequency of 4000 Hz (weighting the loss by age and by gender). The frequency of 4000 Hz is evaluated and the acoustic traumas are classified according to an increasing scale A-B-C-D-E, from higher to lower auditory capacity, assessing the two ears individually [2].

Table 31 shows the sample distribution according to the ELI Index.

**Table 31:** Sample distribution according to ELI Index.

Group	ELI index	Right ear		Left ear	
		# of cases	Frequency %	# of cases	Frequency %
1	A. Normal excellent	590	41.61	477	33.64
2	B. Normal good	271	19.11	285	20.10
3	C. Normal	221	15.59	240	16.93
4	D. Suspected deafness	116	8.18	148	10.44
5	E. Clear indication of deafness	220	15.00	268	19.00
	Total	1418	100	1418	100

#### 2.3.3 Percentage of Global Hearing Loss

This variable has been classified by establishing groups in Percentage of Hearing Loss intervals. This index considers each ear individually (monaaural) and both ears collectively (binaural) [2].

With respect to the Hearing Loss Percentage Index for the Right Ear, the average is a hearing loss of 1.45%, with a minimum value of 0% and a maximum value of 88.13%. In reference to the Hearing Loss Percentage Index for the Left Ear, the average is 1.66%, with a minimum value of 0% and a maximum value of 91.12%. Once it has been discretized and divided into groups. Finally, with respect to the Binaural Percentage Index, the average is 1%, with a minimum value of 0% and a maximum of 67%. Table 32 shows the results obtained.

**Table 32:** Sample distribution according to Hearing Loss Percentage Index.

Group	% Hearing loss	Right ear		Left ear		Binaural	
		# of cases	Frequency %	# of cases	Frequency %	# of cases	Frequency %
1	0	1299	91.61	1256	88.58	1221	86.11
2	≥ 0 <15	70	4.94	103	7.26	163	11.50
3	≥ 15 <30	32	2.26	35	2.47	28	1.97
4	≥ 30 <45	6	0.42	16	1.13	4	0.28
5	≥ 45	11	1	8	1.00	2	0.00
	Total	1418	100	1418	100	1418	100
